# A novel pharmacodynamic assay to evaluate the effects of crystallization inhibitors on calcium phosphate crystallization in human plasma

**DOI:** 10.1038/s41598-017-07203-x

**Published:** 2017-07-31

**Authors:** M. D. Ferrer, M. M. Pérez, M. M. Cànaves, J. M. Buades, C. Salcedo, J. Perelló

**Affiliations:** 1 Laboratoris Sanifit SL, 07121 Palma, Spain; 2 Departament de Nefrologia, Hospital Son Llàtzer, 07198 Palma, Spain; 3 Laboratory of Renal Lithiasis Research, IUNICS, University of the Balearic Islands, 07122 Palma, Spain

## Abstract

Cardiovascular calcification (CVC) is a progressive complication of chronic kidney disease and a predictor of CV events and mortality. The use of biomarkers to predict CV risk and activities of potential or current treatment drugs in these patients could have a crucial impact on therapeutic approaches. Our aim was to develop a novel assay for measurement of the rate of calcium phosphate crystallization in human plasma and provide a tool to evaluate the effects of crystallization inhibitors. The efficacy of inhibitors was determined by adding inhibitory compounds (polyphosphates, fetuin-A, sodium thiosulfate or citrate) to control samples. The assay was additionally validated for SNF472, an experimental formulation of phytate being developed for the treatment of calciphylaxis and CVC in patients with end-stage renal disease (ESRD) undergoing hemodialysis (HD). The method was repeatable and reproducible. The plasma crystallization rate was reduced up to 80% in a concentration-dependent manner following treatment with inhibitors *in vitro*, among which SNF472 was the most potent. This method appears beneficial in evaluating and discriminating between inhibitory activities of compounds such as polyphosphates on calcium phosphate crystallization, which present a novel therapeutic approach to treat CVC in ESRD patients.

## Introduction

Calcification is the normal process of calcium salt deposition in body tissues occurring due to the presence of supersaturated or metastable salt solutions in biological fluids^1, 2^. This process is usually represented by calcium phosphate mineralization in the form of hydroxyapatite (HAP), Ca_10_(PO_4_)_6_(OH)_2_, and controlled by thermodynamic conditions as well as the local concentrations of proteins in biological fluids. This inorganic mineralization mainly occurs in bones and teeth under physiological conditions. However, pathologic ectopic formations, such as calcification in soft tissue and cardiovascular calcification (CVC), are common in calcium-related disorders. CVC is recently described to be not only a passive physicochemical process, as explained above, but also an active cell-mediated process of artery calcification including both atherosclerotic (intimal artery layer) and medial calcification that involves multiple factors and mechanisms with a final common step of ectopic HAP accumulation.

Chronic kidney disease (CKD) and calciphylaxis are disorders that develop as a consequence of disturbances in calcium and phosphate metabolism, and involve inflammatory processes in which levels of circulating calcification inhibitors, such as fetuin-A^3^, matrix-Gla protein^4, 5^, pyrophosphate and osteopontin, are reduced while promoters of calcification are increased. This imbalance between circulating levels of promoters and inhibitors of calcification results in osteogenic transformation and HAP precipitation. In patients with advanced and end-stage renal disease (ESRD), CVC is a normal progressive complication and a predictor of cardiovascular events and mortality^6, 7^. These patients can also suffer from calciphylaxis, resulting in accelerated medial calcification of cutaneous arteries and arterioles^8–11^ and associated with high mortality rates^12, 13^.

Although no approved therapies are currently available for the treatment or reduction of HAP accumulation in CVC, there are evidences from randomized clinical trials of decreasing the progression of CVC in ESRD. These treatments include non-calcium-based phosphate binders to reduce hyperphosphatemia^14^, as well as calcimimetics to treat sHPT^15^. Moreover, other studies demonstrated the efficacy of physicochemical inhibitors of HAP crystal formation, such as bisphosphonates^16, 17^ in the reduction of CVC progression. Therapeutic agents for calciphylaxis additionally include bisphosphonates owing to their anti-inflammatory properties^18^, non-calcium phosphate binders^19^ and sodium thiosulfate (STS), an antioxidant and vasodilator agent^8, 20, 21^. However, none of these compounds have been approved for calciphylaxis and their use is off-label. Several studies have presented evidence supporting the utility of phytate in calcification-related diseases, such as renal calculi^22–24^, osteoporosis^25, 26^ and CVC in animal models^27–30^. SNF472, an intravenous (i.v.) formulation of myo-inositol hexaphosphate (phytate) that binds to the growing sites of HAP crystal, is currently being developed for the treatment of calciphylaxis and CVC in patients with ESRD undergoing HD.

Since calcification is a slow process and no specific biomarkers have been defined to date, evaluation of new therapeutic interventions in clinical trials is an arduous and challenging task. The search for novel biomarkers for effectively assessing the risk of CVC progression has been the major focus of scientific interest in recent years. Assessment of blood crystallization potential in patients with calcium-related disorders may be a relevant parameter to estimate the risk of CVC. Originally, a nanoparticle-based test was developed to measure the propensity for calcification in serum^31^. Subsequent clinical studies demonstrated associations of this nanoparticle marker with CVC and mortality, graft failure after renal transplantation and aortic stiffness^32–34^. However, this method lacked the ability to evaluate the effectiveness of potential therapeutic agents, such as polyphosphates (pyrophosphate, bisphosphonates or phytate). No assays are currently available to assess blood crystallization tendency in the presence of calcification inhibitors. Since polyphosphates protect against crystallization and are the mainstay of CVC treatments^27, 29, 35–37^, the key objective of this study was to develop a novel and rapid *in vitro*/*ex vivo* method to evaluate of calcium phosphate crystallization in plasma samples containing calcification inhibitors and validate its potential as a pharmacodynamic assay for use in both non-clinical and clinical settings.

## Results

### Validation of the spectrophotometric PD assay in human plasma

Intra-day and Inter-day precision were evaluated by calculating %CV and presented as the average of three determinations (Table 1). For both parameters, CV was below 15%, signifying that the assay is both repeatable and reproducible with human plasma samples.

**Table 1:** Intra-day and inter-day precision of the spectrophotometric assay evaluated by determining the average coefficients of variation (CV).

	Slope ± SD^a^ (Abs/logmin)	CV (%)	Mean CV (%)
**Intra-day**			
Assay 1	0.133 ± 0.012	9.1	
Assay 2	0.126 ± 0.011	8.8	7.9
Assay 3	0.143 ± 0.008	5.8	
**Inter-day**			
Day 1	0.090 ± 0.012	13.4	
Day 2	0.108 ± 0.010	9.6	11.9
Day 3	0.108 ± 0.014	12.7	

^a^Mean ± standard error (twelve replicates).

**Figure 1:**
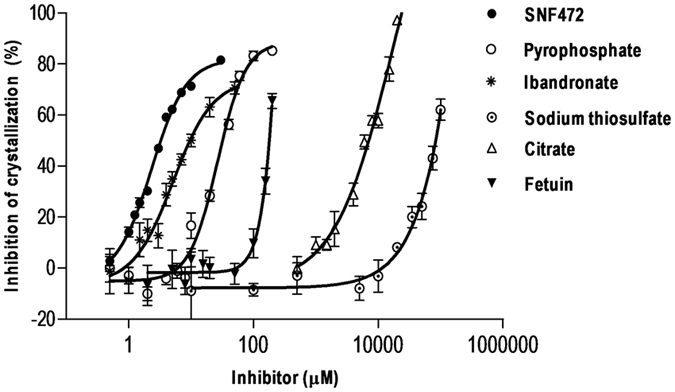
Inhibition of calcium phosphate crystal formation by crystallization inhibitors added *in vitro* to blank human plasma samples. Crystallization was induced by the addition of 12.5 mM calcium and 1.5 mM phosphate, and monitored spectrophotometrically in the linear range between 6 and 24 min. Experiments were performed with six replicates per concentration. Results represent means ± SEM.

Reduction in the crystallization rate in human plasma samples was tested by spiking different concentrations of seven calcification inhibitors into blank plasma samples. As observed in Fig. 1, SNF472 showed the highest potency in inhibiting calcium phosphate crystallization with an IC_50_ value of 2.12 µM. Ibandronate (IC_50_ = 5.13 µM) and pamidronate (IC_50_ = 6.42 µM) were additionally very effective but not as potent as SNF472 (data for pamidronate not shown in Fig. 1 for clarity purposes). Pyrophosphate showed lower inhibitory potency (IC_50_ = 25.97 µM) as well as fetuin (IC_50_ = 545.80 µM). IC_50_ values of citrate and STS were 17.5 mM and >500 mM, respectively.

**Figure 2:**
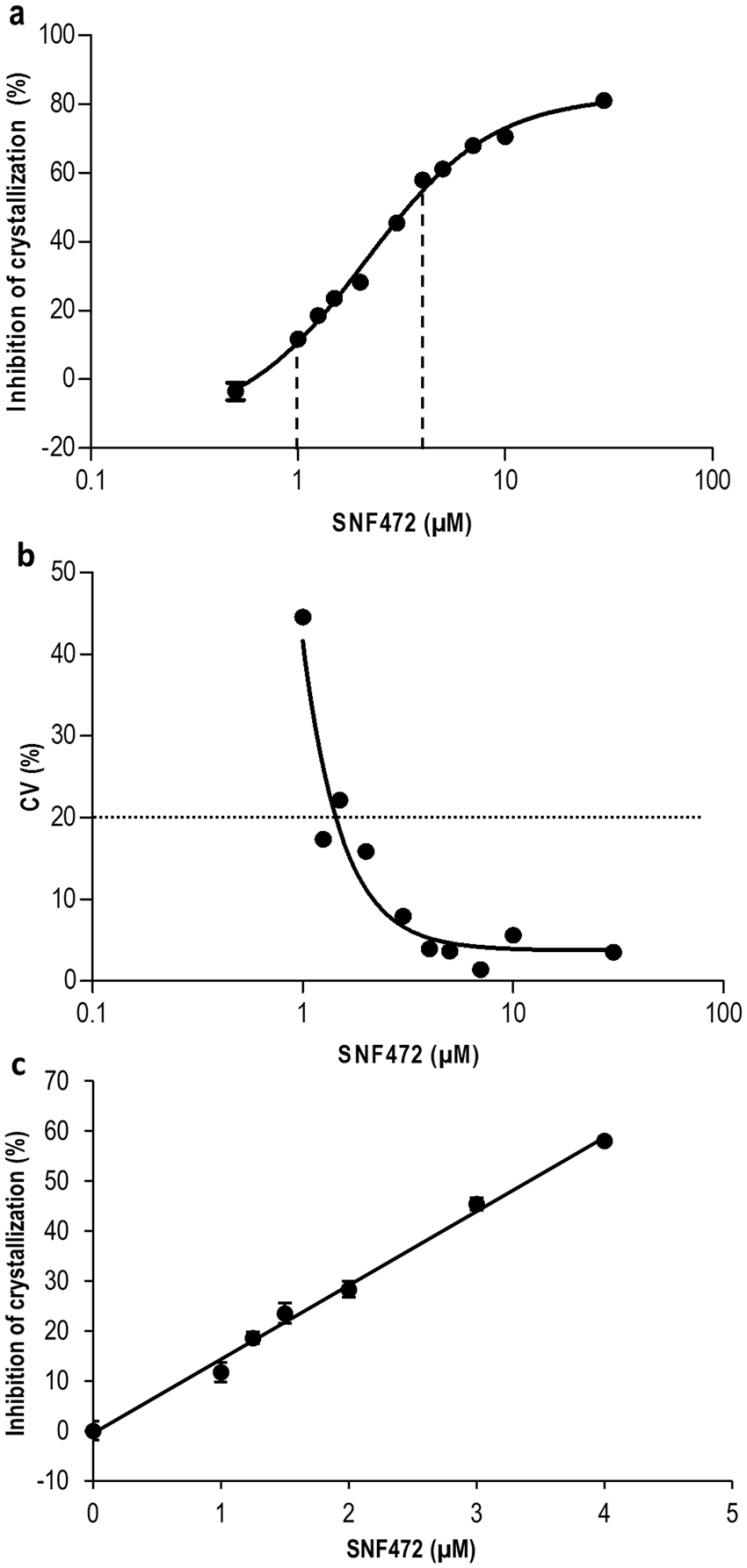
Dose-response curve of the PD assay for SNF472 as a crystallization inhibitor in human plasma samples and precision profile in the range of concentrations tested. (**a**) Dose-response curve. Human plasma samples were spiked with SNF472 in the range of 0.5–30 µM. Crystallization was induced by the addition of 12.5 mM calcium and 1.5 mM phosphate and monitored spectrophotometrically in the linear range between 6 and 24 min. Results are presented as means ± SEM, n = 6. Dashed lines represent the linear portion of the curve (1–4 µM). (**b**) Precision profile. Plot of the coefficient of variation (CV), calculated as the ratio of the standard deviation to inhibition mean, versus the concentration of SNF472 on a log scale. The dotted line represents the initial working range established by a threshold of 20% CV. (**c**) The linear portion of the dose-response curve was used to calculate the lower limit of quantification (LLOQ) for the PD assay in SNF472-spiked human plasma samples (0.77 µM).

**Figure 3:**
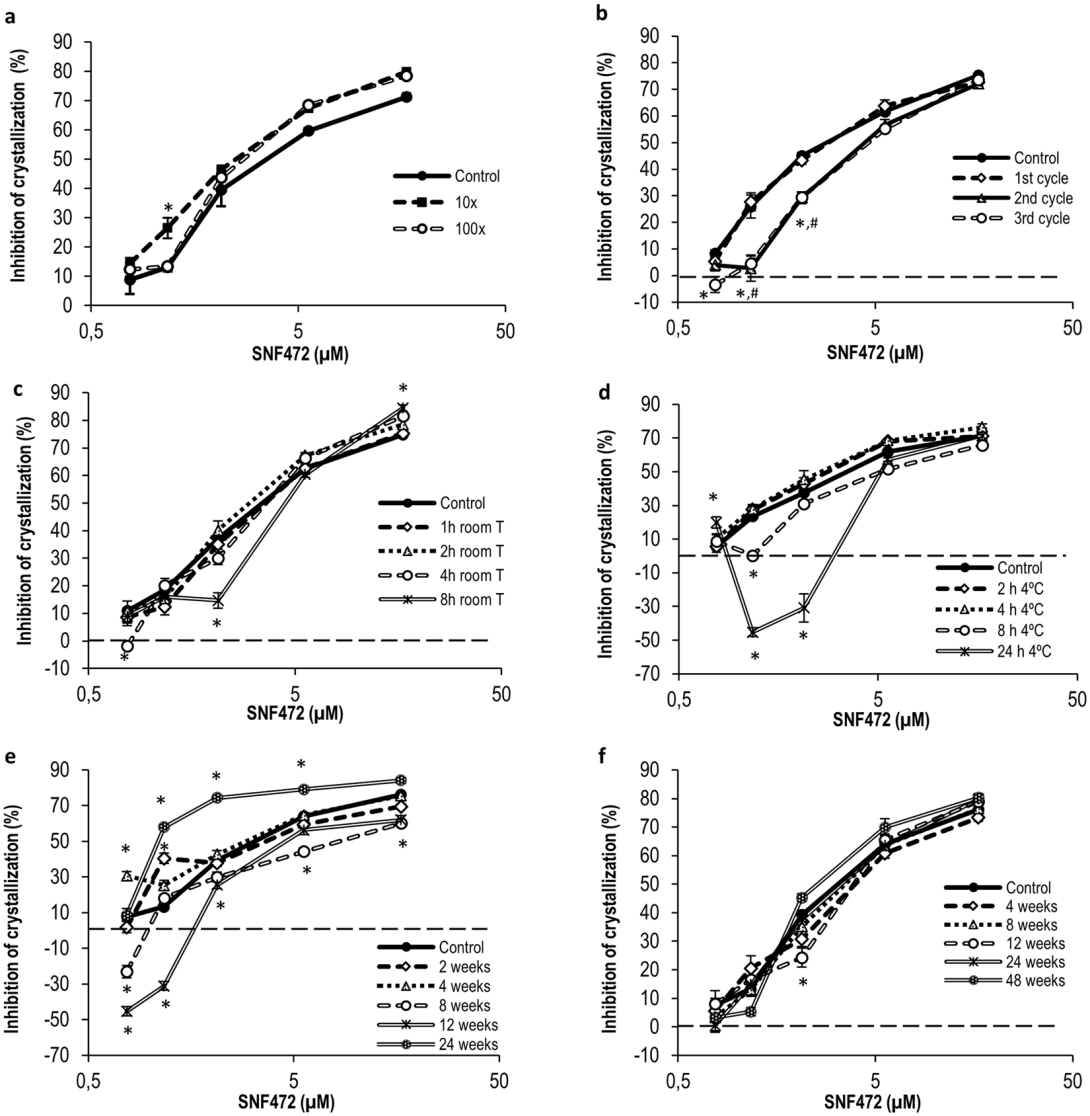
Dilution ability of SNF472 in plasma samples that are above the upper limit of the calibration curve and stability in plasma samples under different storage and temperature conditions. (**a**) *Ex vivo* inhibition of crystallization in undiluted (control) and diluted (1/10 and 1/100) plasma samples (dilution in blank plasma). Results are presented as means ± SEM, n = 6. Statistical analysis (*) of significant differences vs. control samples, P < 0.05. (**b**) Stability of SNF472-spiked plasma samples stored at −80 °C and subjected to one, two or three freeze/thaw cycles. Results are presented as means ± SEM, n = 6. Statistical analysis (*, #) of significant differences vs. control samples, P < 0.05. (**c**) Stability of SNF472-spiked plasma samples maintained for up to 8 h at room temperature. Results are presented as means ± SEM, n = 6. Statistical analysis (*) of significant differences vs. control samples, P < 0.05. (**d**) Stability of SNF472-spiked plasma samples maintained for up to 24 h at 4 °C. Results are presented as means ± SEM, n = 6. Statistical analysis (*) of significant differences vs. control samples, P < 0.05. (**e**,**f**) Stability of SNF472-spiked plasma samples stored at −20 or −80 °C for up to 24 or 48 weeks. Results are presented as means ± SEM, n = 6. Statistical analysis (*) of significant differences vs. control samples, P < 0.05.

To validate the PD assay, the method was applied to evaluate the crystallization inhibition potential in rat and human plasma samples containing SNF472. Linearity, inter-day and intra-day precision, integrity of dilution and stability of phytate spiked in plasma were analyzed. The data obtained for these parameters are presented in Figs 2 and 3 and Table 2.

**Table 2:** Intra-day and inter-day precision of the spectrophotometric assay in determining the efficacy of SNF472 in inhibiting crystallization of calcium phosphate in human plasma.

SNF472 (µM)	Efficacy ± SD (%)	Precision CV (%)
**Intra-day** ^a^		
0.77	9.9 ± 1.4	61.9
1.17	21.1 ± 1.7	17.4
2.12	43.4 ± 1.6	7.2
5.59	68.6 ± 1.9	3.6
16.65	78.3 ± 2.2	3.0
**Inter-day** ^b^		
0.77	9.1 ± 5.1	108.8
1.17	17.2 ± 7.6	38.3
2.12	40.1 ± 7.5	8.7
5.59	64.8 ± 3.8	3.1
16.65	76.8 ± 2.6	2.8

^a^Mean ± standard error (three within-day assays). ^b^Mean ± standard error (three assays in three consecutive days).

A dose-response curve in the defined range (0–30 μM) of SNF472 was obtained, as shown in Fig. 2a. A directly proportional relationship between inhibition of crystallization and concentration of SNF472 was observed within the interval of 1–4 µM (dashed lines) with a correlation coefficient (R^2^) of 0.98. Figure 2b presents the precision profile (CV versus concentration on the log scale) of the method in the calibration range. The dotted line represents the initial working range established by a threshold of 20% CV. A LLOQ value of 0.77 μM was obtained with Eq. (2) applied to the lower portion of the curve defined between 0 and 4 μM SNF472, R^2^ = 0.99 (Fig. 2c). The theoretical concentrations to attain 30%, 50%, 80% and 95% inhibition were calculated via nonlinear regression analysis of the dose-response curve as 1.17 μM, 2.12 μM, 5.59 μM and 16.65 μM, respectively.

The intra-day and inter-day precision of the spectrophotometric assay data used to determine the efficacy of crystallization inhibition in human plasma samples spiked with five different concentrations of SNF472 (LLOQ, IC_30_, IC_50_, IC_80_ and IC_95_) are presented in Table 2. Intra-day precision was low at LLOQ with CV of 61.9%, whereas within-day precision (CV < 20%) was acceptable at concentrations of SNF472 in plasma from IC_30_ onwards, with values between 17.4% and 3.0%. Inter-day results showed low precision at LLOQ and IC_30_ levels (108.8% and 38.3%, respectively), but CV of <20% from IC_50_ onwards.

The ability to dilute samples with concentrations above the upper limit of the calibration curve was assessed (Fig. 3a). Samples diluted 1/10 and 1/100 in blank plasma displayed similar activity values as control non-diluted samples with the same final concentration, with acceptable precision. Data on the stability of SNF472 in plasma samples after storage for different periods of time and under varying temperature conditions are presented in Fig. 3b–f. Samples containing higher concentrations of SNF472 (IC_80_ and IC_95_) were stable after three freeze/thaw cycles. However, significantly lower activities were observed at the IC_30_ concentration in samples exposed to two and three freeze/thaw cycles (Fig. 3b). PD activity did not change in samples maintained for up to 2 h at room temperature, compared to fresh samples as the control (Fig. 3c). Samples were stable for 4 h at 4 °C at all SNF472 concentrations, as shown in Fig. 3d. In addition, samples with SNF472 concentrations around the IC_50_ level or above were stable for up to 8 h at 4 °C while only those with concentrations at the IC_80_ level or above were stable for 24 h under these conditions. Stability of SNF472 spiked in human plasma samples after storage at −20 °C or −80 °C was the last parameter evaluated for assay validation. As shown in Fig. 3e, after four weeks of storage at −20 °C, SNF472 spiked in human plasma remained stable at IC_30_ or higher. Results obtained with samples stored at −80 °C for up to 48 weeks are shown in Fig. 3f. We observed no significant influence of the time of storage at this low temperature on efficacy values.

### Preclinical revalidation of the PD assay in rat plasma

**Figure 4:**
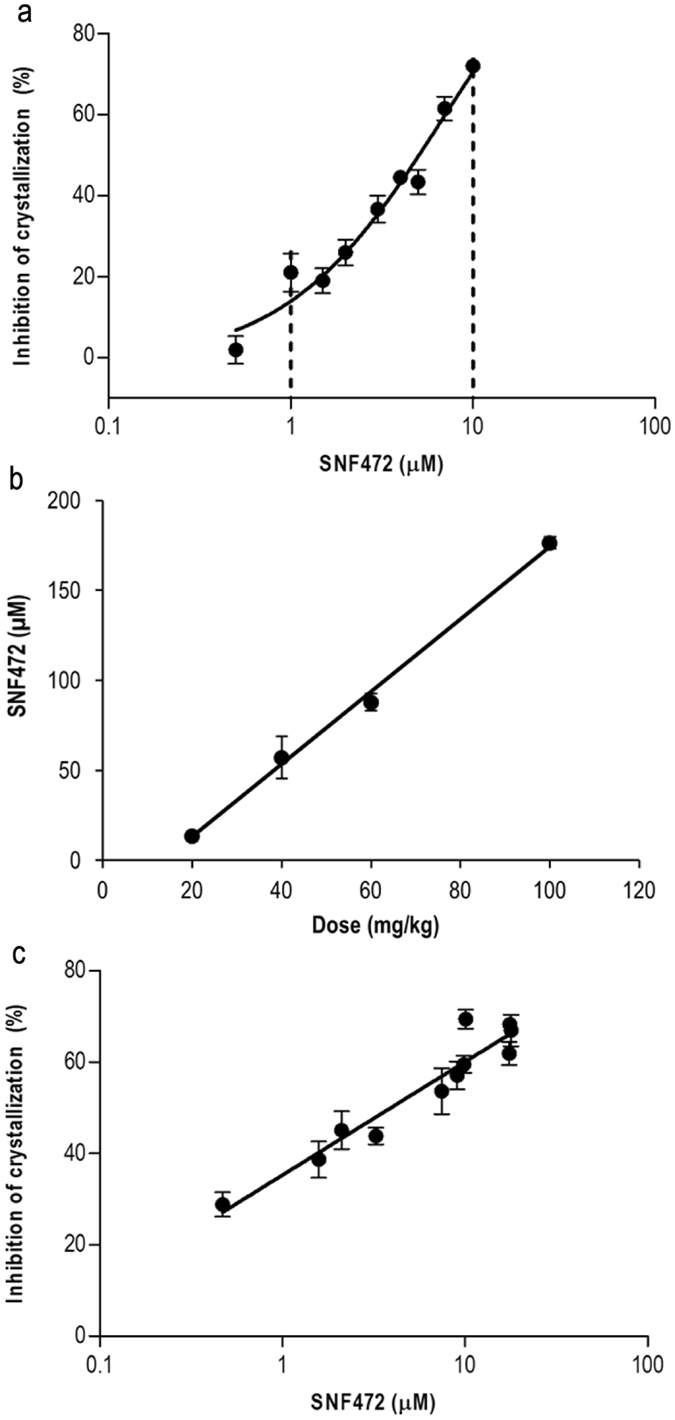
Preclinical validation of the efficacy of crystallization inhibition in rat plasma samples with different concentrations of SNF472 added *in vitro* or *in vivo* and SNF472 plasma levels after 20 min of subcutaneous administration. (**a**) Dose-response curve of the PD assay after *in vitro* addition of SNF472 to rat plasma samples in the range of 0.5–10 µM. Dashed lines represent the interval where inhibition of HAP crystallization is linear in relation to the SNF472 concentration. Results are presented as means ± SEM, n = 8. (**b**) SNF472 levels analyzed using UPLC®-MS in rat plasma samples obtained 20 min after subcutaneous administration. Results are presented as means ± SEM, n = 3. (**c**) Efficacy of SNF472 subcutaneously administered to rats in inhibiting crystallization. Results are presented as means ± SEM, n = 6.

The dose-response curve for SNF472 in rat plasma is shown in Fig. 4a. The linear response of inhibition of calcium phosphate crystals is presented as dashed lines, and observed from 1 up to 10 µM SNF472 (R^2^ = 0.96). As observed in Fig. 4b, total levels of SNF472 in plasma samples obtained 20 min after s.c. injection and analyzed via UPLC^®^-MS were proportional to the administered dose and ranged between a mean of 13.6 μM in rats treated with 20 mg/kg SNF472 and 176 μM in rats treated with 100 mg/kg SNF472. Consequently, rat plasma samples were diluted 1/10 with blank plasma prior to the PD assay to fall within or near the linearity range obtained in the dose-response curve (Fig. 4a). PD results in Fig. 4c showed that s.c. administration of SNF472 to rats effectively reduces crystallization potential in plasma. In addition, inhibition of crystallization was increased in proportion to the SNF472 concentration found in plasma. Table 3 presents the mean SNF472 levels and mean inhibition of crystallization values obtained from the PD assay for each dose administered to rats. Good correlation was observed among doses, plasma levels and crystallization inhibition.

**Table 3:** Inhibition of rat plasma crystallization rate by SNF472 at four different doses quantified after 20 min of subcutaneous administration.

Dose (mg/kg)	SNF472^a^ (µM)	Inhibition of crystallization (%)
0	<LLOQ	0.0 ± 1.9
20	13.6 ± 2.8	37.5 ± 2.6
40	57.2 ± 11.6	44.4 ± 5.3
60	87.8 ± 4.8	56.8 ± 2.0
100	176.4 ± 3.3	65.7 ± 1.6

^a^Mean ± SEM (six replicates).

### Implementation of the PD assay in HD patients

**Figure 5:**
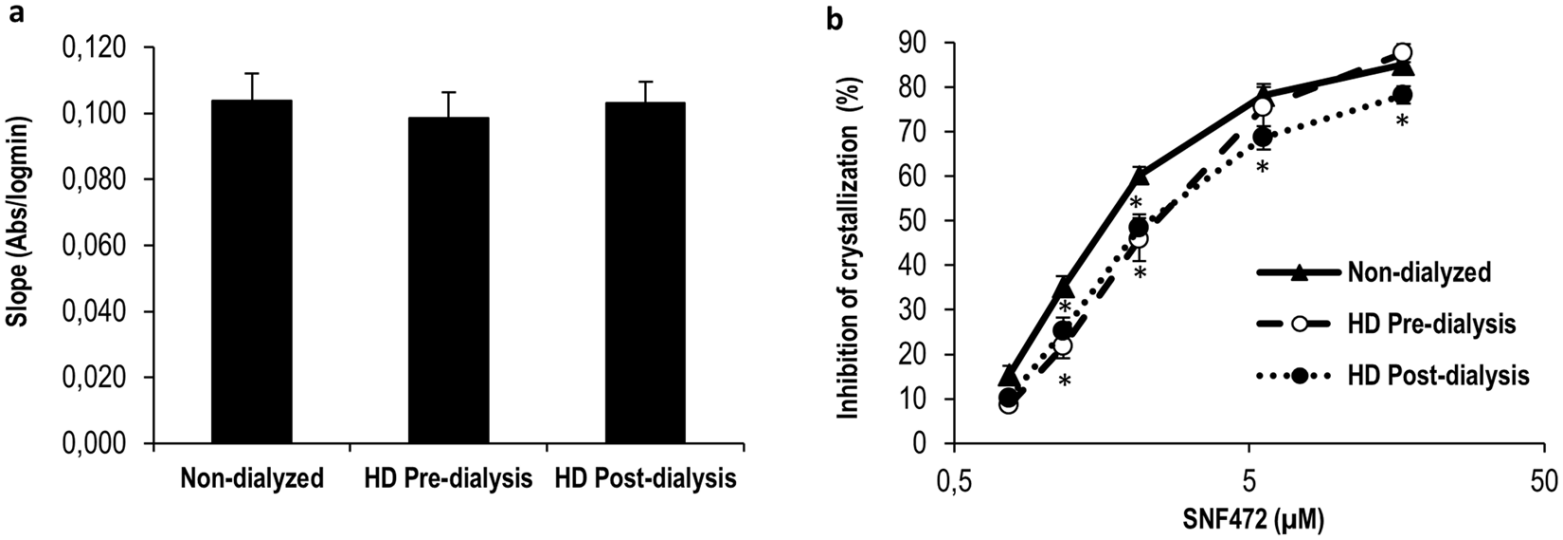
Crystallization potential in blood plasma from pre- and post- dialysis patients and non-dialyzed volunteers before and after *in vitro* addition of SNF472. (**a**) Slopes obtained as a plot of absorbance versus logarithm of time between 6 and 24 minutes. Results are presented as means ± SEM (13 non-dialyzed volunteers and 12 HD patients). (**b**) Dose-response curve of inhibition of crystallization after *in vitro* addition of SNF472 at final concentrations of 0.77, 1.17, 2.12, 5.59, 16.65 µM into plasma. Results are presented as means ± SEM (13 non-dialyzed and 12 HD patients). Statistical analysis was performed using one-way ANOVA. (*) significant differences vs. non-dialyzed healthy volunteers.

As shown in Fig. 5a, plasma obtained from non-dialyzed healthy volunteers and HD patients (pre- and post-dialysis) did not present significant differences in crystallization rate. Data obtained on inhibition of crystallization after *in vitro* addition of SNF472 at final concentrations of 0.77, 1.17, 2.12, 5.59 and 16.65 μM into plasma samples are presented in Fig. 5b. Addition of SNF472 to plasma obtained from non-dialyzed volunteers as well as pre-dialysis and post-dialysis patients reduced the crystallization rate in a concentration-dependent manner (maximum inhibition of 85%, 88% and 78%, respectively). SNF472 suppressed plasma crystallization to a higher extent in healthy volunteers than HD patients at concentrations lower than 5 µM. At higher SNF472 concentrations, similar maximum inhibition rates were estimated in plasma samples from non-dialyzed volunteers and pre-dialysis patients while inhibition in samples from post-dialysis patients was significantly lower.

## Discussion

CVC, a prevalent problem in CKD, is associated with high risk of adverse cardiovascular events and death^38^. Determination of a combination of biomarkers that can be clinically used as a predictor of cardiovascular risk and concomitant therapy for CKD patients is therefore a topic of significant research interest. However, clinical implementation of these markers is limited in some cases, given the need to standardize and validate assay reproducibility. Moreover, clinical applicability of the current cardiovascular biomarkers is more focused on risk of calcification than used as a guide to therapy^39^. Here, we have presented a rapid, repeatable and reproducible *in vitro/ex vivo* spectrophotometric assay that facilitates evaluation of the activities of potential calcification inhibitors of calcium phosphate crystallization in human plasma samples. Our results confirmed the effectiveness of physicochemical inhibitors that target the crystal formation process, such as bisphosphonates^40, 41^, pyrophosphate^42^ and phytate^27–29^ as potential treatments to prevent soft tissue calcification. These are compounds with structural similarities (polyphosphates) that impede further progression of crystallization by binding to HAP crystals and inhibiting vascular calcification. Among the inhibitors tested, SNF472 (an i.v. formulation of phytate) showed the highest potency in inhibiting formation of calcium phosphate crystals in plasma samples (IC_50_ = 2.12 µM). Bisphosphonates and pyrophosphate exhibited IC_50_ values that were in the micromolar range (5.13 and 25.97 µM, respectively), but higher than phytate. These results are consistent with previous studies in which bisphosphonates (non-hydrolysable analogs of pyrophosphate) showed higher efficiency in inhibiting crystallization than pyrophosphate^43^. Moreover, the different crystallization inhibition rates between polyphosphates may be related to the number of phosphates in the chemical structure. While phytate contains six phosphate groups, both bisphosphonates and pyrophosphate contain two groups. Fetuin-A, a well-known circulating serum glycoprotein and potent systemic calcification inhibitor, exhibited lower potency in inhibiting calcium phosphate crystals in the *in vitro* assay (IC_50_ of 545.80 µM). The different inhibitory activities obtained with polyphosphates and fetuin-A may be explained by the fact that polyphosphates present higher binding affinity in the first steps of calcium phosphate formation, while fetuin-A interferes with later steps of the process, including the formation of colloidal (calciprotein) particles, CPP^44, 45^. Citrate does not significantly bind the HAP crystals, but rather chelates calcium. Therefore, greater amounts of this compound are required to start inhibiting crystal formation (IC_50_ 8.000–fold higher than phytate) under these *in vitro* supersaturated calcium conditions. The fact that the calcium concentration is almost 10-fold that of phosphate might explain this lower efficacy shown by citrate. However, it must be noted that the assay is performed in plasma obtained with EDTA, which in fact is chelating part of the added calcium and this might be the reason why calcium concentration had to be pushed so high in order to induce crystallization. Although STS also has the ability to bind free calcium, it is not a strong calcium chelator. In accordance with previously reported *in vitro* observations, we confirm that free calcium is only slightly affected by STS suggesting that STS inhibits vascular calcification at the millimolar range through different mechanisms from both calcium complexation and calcium phosphate crystallization inhibition^46–48^. These collective findings support the utility of our assay as a novel and rapid predictor of crystallization inhibitor activity. This is an important achievement because currently available assays^31^ are not appropriate for use with anionic crystallization inhibitors, such as polyphosphates, since cationic buffers (HEPES) interact with the negative charges of the inhibitors and impede their normal action. Our assay has been optimized for use with NaCl as the matrix and relies on the plasma and phosphate solution to maintain pH near 7.40, facilitating anionic inhibitor activity. Table 4 compares this novel method to those previously developed and that were properly reviewed by Pasch^32^.

**Table 4:** Comparison of the currently available methods to test blood calcification propensity.

	CPP-fetuin-A-test (reviewed in Pasch 2016)	T_50_-test (reviewed in Pasch 2016)	PD-test
**Analytical signal**	Changes in Fetuin-A concentration (mg/l)	T_50_: time of transformation from primary CPPs to secondary CPPs (min)	% Calcium phosphate crystallization inhibition based on speed of crystallization
**Analytical technique**	Ultracentrifugation (16000 × g) and protein quantification	Nephelometry	Spectrophotometry (turbidimetry)
**Particles formed**	CPPs	CPPs	Calcium phosphate crystals (no protein content, no CPPs)
**Temperature**	4 °C	37 °C	Room temperature
**Matrix**	Serum	Serum	Plasma
**Time**	120 min (Ultracentrifugation) 240 min (protein quantification)	600 min	30 min
**Agitation**	No Agitation	No agitation	Orbital plate shaker at 750 rpm
**Media**	—	140 mM NaCl	150 mM NaCl
		10 mM calcium	12.5 mM calcium
		6 mM phosphate	1.5 mM phosphate
		50 mM HEPES buffer	pH = 7.4
		pH = 7.4	
**Modification of test result**	**Human (** ***in vivo*** **):**	**Human (** ***in vivo*** **):**	
	Plasma exchange	HD and hemodiafiltration (−)	
	Cinacalcet	Magnesium Perorally (+)	
	Parathyroidectomy		
	Intradialytic STS		
	HD		
		**Human (** ***in vitro*** **):**	**Human (** ***in vitro*** **):**
		Magnesium (+)	SNF472 (+)
		Calcium (−)	Ibandronate (+)
		Phosphate (−)	Pamidronate (+)
		PPi (−)	PPi (+)
		Fetuin-A (+)	Fetuin-A (+)
		Albumin (+)	Citrate (+)
		Lysozyme (−)	STS (+)
		Gelatin (−)	
		Bicarbonate (+)	
		**Animal (** ***in vivo*** **):**	**Animal (** ***in vivo*** **):**
		Fetuin-A deficient mice (−)	SNF472 (+)

(+) Delay/decrease and (−) increase of the measure.

In view of the efficacy shown by SNF472 and its current development for treatment of CVC and calciphylaxis in ESRD patients, we validated the utility of our assay in obtaining PD clinical measurements for this compound. The linear portion of the calibration curve nearly corresponded to the correct working range (SNF472 > 1.5 μM) at low inhibitor concentrations while at concentrations higher than 4 μM, efficacy reached a plateau and coefficient of variation (CV) was still lower than 20%. Similarly, intra-day precision experiments showed that this *in vitro*/*ex vivo* assay is repeatable with SNF472 concentrations at the IC_30_ value (1.17 μM) or above. In contrast, analysis of inter-day precision indicated that the PD assay is reproducible when inhibitor concentrations are nearly at IC_50_ or higher (2.12 µM). The high variability at low SNF472 concentrations can be explained by the steep response generated by the compound (Hill slope > 1), which makes assessment of its activity below IC_50_ difficult. This is not expected to be a problem, as the final therapeutic levels in humans are expected to be around 14 µM (at T_max_ after intravenous infusion), and therefore PD activity is within the plateau interval where the assay is highly reproducible. If required, SNF472-containing plasma samples can be diluted up to 100-fold in blank plasma to obtain values in the linear range of the assay. In terms of the stability, plasma samples containing SNF472 could be maintained at 4 °C between blood collection and plasma separation. Plasma samples could be successfully frozen at −80 °C over a long storage period (up to one year) and thawed samples maintained at 4 °C for a maximum of 4 h before analysis. The parameters obtained for samples containing SNF472 may be extrapolated to samples containing other polyphosphates.

The validity of the test was confirmed in rat plasma, both *in vitro* and *ex vivo*, as well as in human plasma *ex vivo*. The addition of SNF472 to rat plasma samples reduced the HAP crystallization rate in a concentration-dependent manner up to 80%. Moreover, SNF472 administered subcutaneously to rats suppressed the *ex vivo* HAP crystallization potential of plasma up to 70%. This reduction was dose- and concentration-dependent. Therefore, the plasma concentration of SNF472 reached following s.c. administration has the same maximum efficacy as that *in vitro*, highlighting the utility of our novel PD assay in human studies. Additional non-clinical studies linking the PD assay results with inhibition of cardiovascular tissue calcification should confirm this potential association *in vivo*.

On the other hand, application of the assay to plasma samples obtained from healthy, non-dialyzed volunteers and HD patients revealed no significant differences between the basal plasma crystallization potential of patient and volunteer groups, suggesting that *in vitro* supersaturated conditions lead to loss in sensitivity of the method. This is a significant difference between our new method and the assay previously developed by Pasch *et al*.^31, 32^, which could differentiate the calcification propensity between HD patients and controls. The reason for this discrepancy may lie in the supersaturated calcium and phosphate concentrations used in the current assay. In these conditions, the calcium phosphate crystallization is forced to an extent that higher endogenous calcium or phosphate concentrations might not interfere. Thus, the novelty of our assay relies on its utility in testing the effects of inhibitors on crystallization and comparing their potencies. In this instance, after *in vitro* addition of SNF472 to blood of controls and HD patients, the *in vitro* concentrations required to normalize crystallization potential were higher in HD patients than healthy volunteers, highlighting slight resistance of the blood of HD patients to the inhibitory effects of SNF472, especially at low concentrations. However, maximum inhibition of crystallization (70-80%) could also be achieved in these patients.

In conclusion, we have developed a novel and rapid (30 min) assay to determine the *in vitro*/*ex vivo* inhibition potential of calcium phosphate crystallization inhibitors and validated its efficacy in rat and human plasma samples. Since evaluation of the impact of drug candidates on vascular calcification progression requires long-term clinical trials with hundreds of patients^49^, leading to high research costs, this assay may be effectively employed as a screening tool for rapid evaluation of potential efficacy in suppression of vascular calcification progression. Among the inhibitors tested in the current study, SNF472 (or phytate) was the most powerful, showing 2- and 12-fold higher potency than bisphosphonates and pyrophosphate, respectively. Further validation of results obtained with SNF472 in human samples highlights the potential utility of this assay as a PD measurement tool in the clinical setting.

## Materials and Methods

### A spectrophotometric pharmacodynamics (PD) assay for *in vitro*/*ex vivo* determination of crystallization potential in plasma

The spectrophotometric assay was performed in 96-well plates. Plasma (80 µL) was centrifuged at 10,000 g for 30 min at room temperature and subsequently mixed with 60 µL of 5 mM hydrogen phosphate and 60 µL of 41.67 mM calcium to attain final concentrations of 1.5 mM phosphate and 12.5 mM calcium, respectively. All reagent solutions were filtered and pH adjusted to 7.4. Crystallization of calcium phosphate was monitored spectrophotometrically for 30 min at room temperature via increase in absorbance at 550 nm using the Biotek Powerwave XS Microplate spectrophotometer. The plate was incubated at room temperature in an orbital shaker (750 rpm) and absorbance measured every 3 min. Plasma crystallization potential was assessed based on slope measurement in the linear range between 6 and 24 min from plots of increase in absorbance versus logarithm of time.

### Validation of the spectrophotometric PD assay in human plasma

#### Sample collection

This study was conducted according to the guidelines of the Declaration of Helsinki. All procedures involving human subjects/patients were approved by the Ethical Committee of Clinical Investigation of the Balearic Islands (IB 2245/14 PI). Participants were informed of the purpose and characteristics of the study before providing written consent.

Venous blood samples were obtained from the antecubital vein of healthy volunteers under fasting conditions and collected in tubes with K_2_EDTA as anticoagulant. Plasma was obtained after centrifugation of blood at 3,500 rpm at 4 °C for 10 min and mixed to form a pool.

#### Intra-day and inter-day precision under repeatability and reproducibility conditions

The spectrophotometric PD assay was repeated with three runs in the same day (intra-day precision) and three consecutive days (inter-day precision) in a 96-well plate (12 replicates per run). Intra-day precision refers to the repeatability of the assay while inter-day precision indicates between-day precision (reproducibility). Both parameters were evaluated by determining the within-day and between-day average coefficients of variation (CV) of the slopes obtained.

#### Inhibition of HAP crystallization in plasma samples

The efficacy of different calcification inhibitors in preventing *in vitro* formation of calcium phosphate crystals was assessed in human plasma samples using the spectrophotometric PD assay. Inhibition of crystallization was measured by comparing the slopes of the control sample (blank) with those of samples containing the crystallization inhibitor as shown below:

1 $$ \% \,\mathrm{Inhibition}\,\mathrm{of}\,\mathrm{crystallization}\,=\frac{\mathrm{slope}(\mathrm{blank}\,\mathrm{plasma})\,-\,\mathrm{slope}(\mathrm{inhibitor})\,}{\mathrm{slope}(\mathrm{blank}\,\mathrm{plasma})\,}\cdot \mathrm{100}\,\,$$

Human plasma was spiked with increasing concentrations of inhibitors. Solutions of inhibitors were prepared by dilution in 0.15 M NaCl and pH adjusted to 7.40. The inhibitors and final concentration ranges tested in plasma were as follows: SNF472 (0–30 μM), pyrophosphate (PPi, 0–200 μM), ibandronate (0–50 μM), pamidronate (0–50 μM), STS (0–100000 μM), citrate (0–25000 μM) and fetuin-A (0–208 μM). To compare the potency of inhibition, half-maximal inhibition concentrations (IC_50_) were calculated using GraphPad Prism 5.0 software.

### Validation of the spectrophotometric PD assay in determining the efficacy of SNF472 on inhibition of crystallization in human plasma

The spectrophotometric method was validated through evaluation of linearity, intra- and inter-day precision, integrity of dilution and stability of plasma samples spiked with SNF472 under long-term (−20 and −80 °C) and benchtop (room temperature and 4 °C) conditions and following freeze/thaw cycles.

Linearity was evaluated using solutions of SNF472 to attain final concentrations of 0.5, 1.0, 1.25, 1.5, 2.0, 3.0, 4.0, 5.0, 7.0, 10 and 30 µM in plasma. The calibration curve generated in the linearity assay was applied to calculate the lower limit of quantification (LLOQ), IC_30_, IC_50_, IC_80_ and IC_95_ values. LLOQ was determined using Eq. (2) and IC_30_, IC_50_, IC_80_ and IC_95_ values calculated with GraphPad Prism 5.0 software.

2 $$\mathrm{LLOQ}=\mathrm{10}\times \frac{\mathrm{Standard}\,\mathrm{deviation}\,\mathrm{of}\,\mathrm{the}\,\mathrm{response}}{{\rm{Slope}}}$$

The assay was repeated with three runs per day for three consecutive days (12 replicates for LLOQ, EC_30_, EC_50_, EC_80_ and EC_95_ of SNF472 in plasma per run) to assess intra-day and inter-day precision, respectively, under repeatability and reproducibility conditions. Repeatability and reproducibility of the PD assay were evaluated by determining intra- and inter-day CV.

The ability to dilute samples that were originally above the upper limit of the calibration curve was studied by diluting SNF472-concentrated samples in blank plasma. LLOQ, EC_30_, EC_50_, EC_80_ and EC_95_ values of SNF472 in plasma per dilution factor (10x and 100x) were assayed in one run with six replicates.

The stability of SNF472 in plasma samples was evaluated at final LLOQ, IC_30_, IC_50_, IC_80_ and IC_95_ concentrations in plasma. Plasma samples were immediately assayed in one run with six replicates before and after storage for different time-periods and temperature conditions. For long-term storage, plasma samples were frozen at −20 or −80 °C after SNF472 spiking and stored between 2 weeks and 12 months. In terms of benchtop stability, plasma samples were stored on the bench at room temperature between 1 and 8 h or at 4 °C between 2 and 24 h. For freeze/thaw cycles, plasma samples were frozen at −80 °C, thawed and refrozen up to three times in consecutive days. Once completely thawed, plasma samples were used for assay or refrozen for a further freeze/thaw cycle.

### Preclinical revalidation of the PD assay in rat plasma samples

A preliminary assay was performed *in vitro* by adding increasing concentrations of SNF472 to blank rat plasma to obtain the linear range for inhibition of crystallization and calculate LLOQ and IC_50_ values. Blood from three Sprague-Dawley male rats (282 ± 4 g, Charles River Laboratories, France) was collected in tubes with K_2_EDTA as anticoagulant. Plasma was obtained after centrifugation at 3,500 rpm for 10 min at 4 °C and mixed to obtain a pool. Nine SNF472 concentrations were prepared within a range of 0.5 to 10 µM.

In the second assay, five groups of three male Sprague-Dawley rats (314 ± 4 g, Charles River Laboratories, France) were subcutaneously administered 0 (saline), 20, 40, 60 or 100 mg/kg SNF472. Blood was collected after 20 min in tubes with K_2_EDTA, and plasma separated via centrifugation at 3,500 rpm for 10 min at 4 °C. The crystallization potential of plasma samples was measured using the spectrophotometric PD assay.

In parallel, SNF472 levels in the same rat plasma samples were determined by means of phytic acid quantification. Phytic acid was quantified via LC-MS using a method previously described by Tur *et al*.^50^.

### Implementation of the PD assay in HD patients

#### Sample collection

Experiments were conducted according to the guidelines of the Declaration of Helsinki. All procedures involving human subjects/patients were approved by the Ethical Committee of Clinical Investigation of the Balearic Islands (IB 2245/14 PI). All participants were informed of the purpose and characteristics of the study before providing written consent.

A total of 13 healthy volunteers and 12 ESRD patients undergoing HD treatment in Hospital Son Llàtzer (Palma, Spain) participated in the study. One (volunteers) or two (HD patients) venous blood samples were obtained from the antecubital vein (volunteers) or the afferent line (using the sample port in HD patients), and collected in tubes with K_2_EDTA as anticoagulant. Two blood samples were obtained from HD patients before and after dialysis treatment. Blood was centrifuged at 3,500 rpm at 4 °C for 10 min to separate plasma.

### Assessment of crystallization potential in plasma samples obtained from healthy volunteers and HD patients

The PD assay was performed on blank plasma samples and after *in vitro* addition of variable concentrations of SNF472 to attain LLOQ, IC_30_, IC_50_, IC_80_ and IC_95_ values of SNF472 in plasma.

### Statistical analysis

Results are expressed as means ± SEM. Analyses were performed using the statistical software package GraphPad Prism 5.0. Statistical significance of data was analyzed using two-way ANOVA. In cases where significant effects were observed, a post-hoc Bonferroni test was applied. *P*-values < 0.05 were considered statistically significant.
